# Quantifying the Impact of Grain for Green Program on Ecosystem Service Management: A Case Study of Exibei Region, China

**DOI:** 10.3390/ijerph16132311

**Published:** 2019-06-29

**Authors:** Qianru Yu, Chen-Chieh Feng, NuanYin Xu, Luo Guo, Dan Wang

**Affiliations:** 1 College of Life and Environmental Sciences, Minzu University of China, Beijing 100081, China; 2 Department of Geography, National University of Singapore; Singapore 117570, Singapore; 3 School of Urban Planning and Design, Peking University, Shenzhen 518055, China; 4 International Doctoral Innovation Centre, Department of Chemical and Environmental Engineering, University of Nottingham Ningbo China, Ningbo 315100, China

**Keywords:** ecosystem services, grain for green program, scarcity analysis, land use change, exibei region

## Abstract

Evaluating the impact of an ecological restoration program on ecosystem services is crucial, given the role of such a program in boosting sustainable ecosystem management. This study examines the impact of one of the large-scale ecological restoration programs in China, the Grain for Green Program (GGP), on ecosystem service management in the Exibei region of China. This region is studied, as it is a key source water area with rich biodiversity and has been experiencing GGP for 20 years. To achieve the stated goal the changes of land use and ecosystem services value (ESV) and the ecosystem services scarcity value (ESSV) in the Exibei region were quantified and assessed based on remote sensing images from 1990, 1995, 2000, 2005, 2010, 2015 and field survey data. The results indicated that the expansion of construction land and the increase of water body were the dominant land use changes throughout the study period. Farmland, forestland and grassland decreased by 2.61%, 0.47% and 1.41% after the GGP, respectively. The ESV of the entire Exibei region increased slightly in response to land use change during 1990–2015, with an annual loss of 0.08% before the implementation of GGP and an annual growth of 0.03% after the implementation of GGP. Moreover, forestland was the dominant contributor to ESSV after the implementation of the GGP. Its annual growth rate was four times higher than before the commencement of GGP. The results of this study contribute to the protection of the Exibei region ecosystem, and more importantly, the future management of the ecosystem service in the hilly regions of southern China.

## 1. Introduction

Ecosystem service refers to the set of environmental conditions and products by ecosystems in the ecological process [1,2,3], which the ecosystem offers to benefit people [4,5]. It is also an indicator that reflects the healthiness of the ecosystem [2,6]. Maintaining a sustainable ecosystem is therefore crucial, not only because of the services the ecosystem, provides but also because people may unknowingly cause irreversible impacts on the ecosystem. Quantifying the impacts of human activities on ecosystem services is an indispensable step to ensure the sustainability of an ecosystem and the ecosystem services provided. 

Over the past two decades, satellite-based assessments of ecosystem services (ES) have been found useful by natural resources managers, and especially when setting conservation priorities [7,8,9,10]. There are three main approaches to assessing ecosystem services: (1) biophysical methods for mapping ecosystem services; (2) a monetary valuation approach for estimating that ecosystem services; and (3) socio-cultural methods for understanding preferences or social values of ecosystem services [9]. Among the three methods, monetary valuation has been widely used because it facilitates faster assessment [11] and it is an effective way to communicate to policymakers, business sectors, and the general public to realize the importance of environmental conservation [12]. Therefore, monetary valuation was used to assess ecosystem services in this article.

Environmental policies and decisions have a clear influence over managing an area, which in turn affect the status and integrity of the ecosystems there. These relationships highlight the need to understand how land use plans affect sustainable natural resource management [13,14]. Thus, ecosystem service assessments are widely used in land use policy and management practices [5,15]. Several countries have implemented large-scale ecological restoration projects to improve regional ecosystem services [5]. The Grain for Green Program (GGP) is a natural ecological system restoration program of China. It is also the largest forest ecological construction project in the world [16,17,18]. The program was launched in 1999 with a few pilot studies and expanded into most of China in 2002 [19]. It has been run continuously for two decades. It aims to increase vegetation coverage and reduce soil erosion by converting existing farmland to forestland or grassland on steep slopes or severe desertification farmland [20]. To ensure its acceptability by the local farmers and its sustainability, the program uses a compensation scheme to encourage local farmers to voluntarily convert the land units on steep slopes or severe desertification farmland used for planting crops to planting trees or grass [21,22]. The GGP has so far been adopted by 32 million households in 25 provinces and returned 15.31 million hectares of marginal cropland to forest and grassland [23,24].

The Exibei region, located in the middle reaches of the Yangtze River in China, is a crucial area providing an ecological barrier for combating desertification. As the extensive deforestation contributed to severe water and wind erosion of soil during the 1970s to 1990s, in particular, the devastating 1998 flood in the Yangtze River basin [25], the Exibei region was one of the earliest candidate sites for the implementation of GGP. This location therefore would serve as a good case study to assess how land use management may impact ecosystem services.

This study aims to identify the impacts of GGP on the ecosystem services in the Exibei region. Using remote sensing techniques, land use changes in the study area between 1990 and 2015 were first quantified. Tupu, a methodology for spatial and temporal graphical analysis [26,27], was used to analyze land use changes. The most evident requirement of quantitative land use changes is not only the area change. As the geographic patterns of land use changes are equally important [28], the study also quantified such geographical patterns. Using the market-valuing method [29,30] and socio-economic-natural complex ecosystem theory, the natural subsystem and socio-economic subsystem of the study area were organically combined through value accounting. A sensitivity analysis was run to ensure the accuracy of ecological value coefficients, and the variable coefficient was calculated to quantify the change tendency of land use types. The scarcity value of ecosystem services, which measured the infinite desire of human beings versus the limited resources that satisfy human needs in economics [31], was calculated to quantify the need for ecosystem services with the GGP. By analyzing the changes in ecosystem service values and land use, this study is helpful to provide policy suggestions for comprehensive management and sustainable development for implementing the GGP in China and other regions with similar ecological restoration projects.

## 2. Materials and Methods 

### 2.1. Study Area

**Figure 1:**
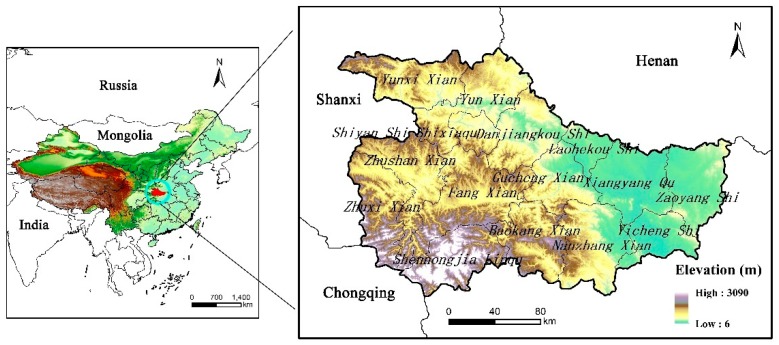
The location of study area.

Located in the northwest of Hubei province in China, the Exibei region lies approximately between 31.22° to 33.27° N latitude and 109.48° to 113.1° E longitude, covering an area of 49,340 km^2^ (Figure 1). The region has a subtropical monsoon climate characterized by four seasons and abundant precipitation and sunshine. It is therefore an important water management basin for the Han River. Mountains account for roughly 65% of the total area. The main mountains extend in the northwest-southeast direction and are part of Qinling and Dabashan mountain ranges, with the elevations between 90 to 3100 m above mean sea level. The middle reach areas of the Han River pass through the region, with branches forming a lattice drainage pattern. The Exibei region is the essential water conservation area and its forestland is the dominant type, with high forest coverage of more than 64%. The region has a population of 0.91 million people in 15 counties. Its population density is 1.27 times higher than the national average.

### 2.2. Data

In the Exibei region, the pilot project of the GGP Program was carried out in some counties and districts in 2000. In 2001, the GGP was officially launched in the whole region. In order to compare the land use changes in a decade before and after the implementation of the GGP in the Exibei region in 2001, the land use data of study area of 1990, 1995, 2000, 2005, 2010 and 2015 were used. This dataset was obtained from the Chinese Academy of Sciences Resource Environmental Data Center (RESDC) [32], which was produced based on Landsat TM−/ETM+, Landsat8 OLI and GF-2 [33,34], and a semi-automated approach via human-computer interaction as described in Liu et al. [33] to classify the input data into land-use types intended for the development of a national land-use databases of 1990, 1995, 2000, 2005, 2010 and 2015. The classification and overall accuracies were ultimately evaluated through the confusion matrix. The comprehensive evaluation accuracy of the first level of land use is at least 93% and that of the second level is at least 90% [33,34], which meets user mapping accuracy demands at a scale of 1:1 million [34]. We divided the land-use in the study area into six primary and 17 subtypes according to the National Standard Land-Use Classification of China. The six first levels of land use types include farmland, forestland, grassland, water body, construction land, and unused land. Net primary productivity (NPP) data were obtained from RESDC as well. The social and economic data was derived from China Statistical Yearbook [35], China Statistical Yearbook on Environment [36], and Hubei Province Statistical Yearbook [37]. The data for semi-structured interviews were conducted in August 2018 through a field survey.

### 2.3. Methods

#### 2.3.1. Geo-Information Tupu Change Analysis Based on GIS

Geo-information Tupu provides an effective way to study land use pattern and process integration [38] due to its capability to describe the development process of the land-use types, and to show its internal rules [39]. It combines the characteristics of spatial units (i.e., Tu) and the starting point of processes and events (i.e., Pu) in one common framework to shed light on the development and change the rule of geographic phenomena [26]. 

In order to build the series of Tupu models [40] of land-use change in the Exibei region, the land-use data of the aforesaid six periods were conducted with map algebraic superposition by ArcGIS (Environment System Research Institute, Redlands, CA, USA) following the formula described in [27], shown below:

(1) $$T=Y_{1}\times 10^{n-1}+Y_{2}\times 10^{n-2}+\cdot \cdot \cdot +Y_{n}\times 10^{n-n}$$

Wherein, *T* is the Tupu unit code value of the Tupu mode characteristics within the representation research stage; *Y_n_* is the land use Tupu unit code value of representation in some year; *n* is the number of land use type. 

Tupu codes refer to transition type of land use changes. For example, a Tupu code of *AB* means that land use unit code value of the previous stage of representation is *A* and the land use unit code value of the later stage of representation is *B*, and the land use type had converted from A to B. In order to express the change of characteristics of land-use pattern explicitly, the spatial separation index of land use change [26] is introduced. The formula of change ratio and spatial separation degree as follows:

(2) $$R_{ij}=Aij\times 100\%/\overset{n}{\underset{i=1}{\sum }}\overset{n}{\underset{j=1}{\sum }}A_{ij}$$

(3) $$S_{ij}=\frac{1}{2}\times \sqrt{D_{ij}/\overset{n}{\underset{i=1}{\sum }}\overset{n}{\underset{j=1}{\sum }}A_{ij}}/A_{ij}/\overset{n}{\underset{i=1}{\sum }}\overset{n}{\underset{j=1}{\sum }}A_{ij}$$

Wherein, *R_ij_* is Change ratio. It presents the ratio of the Tupu unit area of individual land use change to the total area of all intra-regional transferred land use change Tupu. *S_ij_* is Space separating degree, and reflects the dispersion degree of land use Tupu unit in spatial distribution. The more the desperation degree value, the less aggregation of Tupu unit in spatial distribution. *D_ij_*, *A_ij_* presents the number of Tupu unit and area of land use type *i* of t at initial stage changes to land use type *j* of )*t +* *Δt*) at a later stage, and *n* is the number of land use type.

#### 2.3.2. Measuring Ecosystem Services Value

This paper adapts the method of Costanza et al. [4] and the study results of Xie et al [30] to calculate the ecosystem service value (ESV) and its change in the Exibei Region between 1990 and 2015. The ESV estimation method by Constanza et al. [4] was well received and used widely. However, due to the uniqueness of China’s ecosystem, their approach must be adapted for ecological studies in China. Xie et al. [30] advanced the work of Costanza et al. [4] by surveying more than 700 professionals in ecology in China in 2003 and 2008. 

Xie et al. [30] set the coefficient for annual natural food production function of farmland by natural ecological processes per hectare per year was assigned as 1, which is 1/7 of the actual food production value [11]. Taking into account the reality in the study area and the ease of data acquisition, we used the average actual food production data from farmland per hectare per year and the market crop prices from 2010 to calculate the ESV. In the Exibei region, the annual average of actual food production from farmland in 2010 was 6119.88 kg/ha, and the annual average actual food production in China was 4974.00 kg/ha in 2010. The economic value equivalent of food production in China in 2010 was 3406.50 yuan/ha (or US$503.21 ha in 2010), and the average ESV of one equivalent value for the Exibei region was calculated as 4191.27 yuan/ha (or US$618.95 ha in 2010).

Although climate, social and economic factors all affect ESV, in order to determine the impact of land use on this value, this study assumed that these factors assert effects that are negligible. This is because the climate and socio-economic conditions remained more-or-less the same over the period of study. The ESV was calculated using the following valuation equations [41].

(4) $$ESV=\sum \left(A_{n}\times VC_{n}\times R\times W\times A\right)$$

(5) $$W=2/\left(1+e^{-m}\right)$$

(6) $$m=1/\left[En_{r}\times \left(1-P_{u}\right)+En_{u}\times P\right]-2.5$$

Wherein, *ESV* was the ecosystem services value; *A_n_* was the area of land use type *n*-th (ha) and *VC_n_* is the ecosystem services value per unit area of land use type *n*-th in China (yuan/ha). *R* was the correction coefficient of *VC_n_*, which was the ratio of *NPP_e_* to *NPP_c_*; *W × A* was the socio-economic adjustment coefficient; *W* was the willingness to pay for *ESV* and could be calculated by the logistic regression model; *A* was the ability to pay for *ESV* and could by calculated by gross domestic product (GDP) per capita. *NPP_e_* was the net primary productivity of natural vegetation in Exibei; *NPP_c_* was the average net primary productivity of natural vegetation in China. *En_r_* and *En_u_* respectively was Engel coefficient of rural and urban area in 2010; *P_u_* was the percentage of urban population in 2010 (%). *GDP_e_* was per capita GDP of study area in 2010 (yuan/person), and *GDP_c_* was per capita GDP of China in 2010 (yuan/person).

#### 2.3.3. Measuring Ecosystem Services Scarcity Value

Ecosystem services are affected by human interests. As they enjoy ecosystem services, these ecosystem services become scarce [42]. In this study, an economic conceptualization was used where simultaneous changes in supply and demand influence ecosystem services value via their effect on relative scarcity. Both the supply curve and the demand curve were assumed to be linear, and ecosystem services were classified broadly as either private or public goods [4,43]. Demand for private-good ecosystem services is typically characterized by a steep slope, or inelastic (i.e., price elasticity of demand <1), and the price elasticity of supply for them is characterized by a gentler slope, or elastic (i.e., price elasticity of supply >1). The converse holds true if ecosystem services are public goods [44]. Following Bryan et al. [45], we developed three scenarios of simultaneous changes in supply and demand in three scenarios and calculated the value of scarcity. Simultaneous change in supply and demand over time changes the per-unit scarcity value of ecosystem services is represented here as price, and scaled the quantity demanded and supplied *Q_0_* and the price *P_0_* at the 1990 equilibrium point to both equal 1 (Table 1).

(7) $$ESV=\sum ESV_{n,t}=A_{n,t}\times VC_{n,t}\times \left(1+\Delta p_{n,t}^{Sup}+\Delta p_{n,t}^{Dem}\right)$$

Wherein, *A_n_* was the area of land use type *n*-th (ha) at time t, and *VC_n_*, t was ecosystem services value per unit area of land use type *n*-th in China (yuan/ha) at time *t*. *Δp^Sup^_n,t_* was the supply-driven relative change in scarcity value, and *Δp^Dem^_n,t_* was the demand-driven relative change in scarcity value.

(8) $$\Delta p_{n,t}^{Sup}=\Delta Q_{n,t}^{Sup}\times \Delta P_{n,t}^{Sup}$$

(9) $$\Delta Q_{n,t}^{Sup}=-\frac{\sum ESV_{n,t1}\times \sum ESV_{n,t0}}{\sum ESV_{n,t0}}$$

The change in supply *ΔQ^Sup^_n,t_* of each ecosystem service was proportional to the change in the physical service value of supplied between *t0* and *t1*.

(10) $$\Delta p_{n,t}^{Dem}=\Delta Q_{n,t}^{Dem}\times \Delta P_{n,t}^{Dem}$$

(11) $$\Delta Q_{n,t}^{Dem}=\left(WTP_{n,t1}-WTP_{n,t0}\right)/WTP_{n,t0}$$

(12) $$WTP_{n,t}=POP_{t}\times GDP_{t}\times \varepsilon_{n,t}$$

The change in demand *ΔQ^Dem^_n,t_* from *t0* to *t1* was then calculated as the proportional change in the willingness to pay of the Exibei population.

Wherein, the willingness to pay was calculated as a function of the total population (*POP*) at time *t*, wealth measured as the real gross domestic product (*GDP*) per capita at time *t* adjusted for inflation to 2010 yuan, and the income elasticity of demand *ɛ_n,t_* for each ecosystem service *n* and year *t*, we used published income elasticity estimates directly applicable to specific ecosystem services.

#### 2.3.4. Sensitivity Analysis and the Coefficient of Variation Analysis

In this study, a sensitivity analysis using the standard economic concept of an elastic coefficient was used to assess the degree of dependence of ESV coefficient, with the change of time had on the ESV. The sensitivity coefficient validates the accuracy of ecological value coefficients. The calculation equation is as follows [46]:

(13) $$CS=\left|\frac{\left(ESV_{j}-ESV_{n}\right)/ESV_{n}}{\left(VC_{jk}-VC_{nk}\right)/VC_{nk}}\right|$$

Wherein, *ESV*, *VC*, and n are the same variables as used before, and i and j are the parameters before and after adjustment, respectively. If *CS* > 1, then *ESV* is elastic with respect to ecosystem value coefficient, and the accuracy is correspondingly low; If *CS* < 1, then *ESV* is inelastic with respect to the ecosystem value coefficient, and the accuracy of ecosystem value coefficient and the estimation of ecosystem service value is more accurate.

The coefficient of variation can be used to express the differences in ecosystem service value among the different land use type in the Exibei region. This is accomplished using the following formula [1,47]:

(14) $$CV_{j}=S_{j}/Y_{j}=\sqrt{\frac{1}{n-1}\overset{n}{\underset{i=1}{\sum }}{\left(Y_{ij}-Y_{j}\right)}^{2}}/\left(\frac{1}{n}\overset{n}{\underset{i=1}{\sum }}Y_{ij}\right).\;$$

Wherein, *V_j_* is the coefficient of variation, *S_j_* is the standard deviation of ecosystem services value of land use type at time *j*, *Y*_j_ is the average of ecosystem services value of land use type at time *j*, *Y_ij_* is the ecosystem services value of land use type *i* at time *j*, and *n* is the number of land use types.

#### 2.3.5. Field Survey and Semi-Structured Interviews

Ecosystem provides multiple ecosystem service benefit humans, and humans can also feel the change of ecosystem services directly. It is important to integrate local resident awareness and perception of ecosystem services (ES) into the ES assessment with the implementation of GGP. We conducted a semi-structured interview with the local residents. Since ES are assessed comparatively, it is critical that this evaluation is carried out within a finite set of services related to a given geographical area, so that all the respondents make a judgment from the same choice set and in the same context [48].

A semi-structured interview through a standard questionnaire was used to obtain demographic characteristics and preference of ecosystem services pertinent to people’s life [49]. Interviews focused on the following themes: personal background, general perceptions of the neighborhood, environmental perceptions and agency [50]. The 45 interviewees from the four villages participating in the GGP were asked of their preference of 11 ecosystem services. If necessary, illustration and explanation of each ecosystem services were provided to ensure that the interviewees understood the questions. The interviews were undertaken in a conversational style with questions used as prompts to ensure the respondents provide as much information as possible. Each question was eventually assigned rating scale, e.g., very satisfied, satisfied, neutral, not so satisfied and very unsatisfied. Transcription of notes was done as quickly as possible after each interview. Based on these interviews, the perceived in ecosystem services was created. The interpretation and illustration were used in objective to avoid bias in interviewees, following [51].

## 3. Results

### 3.1. Land Use Change Based on Geo-Information Tupu 

**Figure 2:**
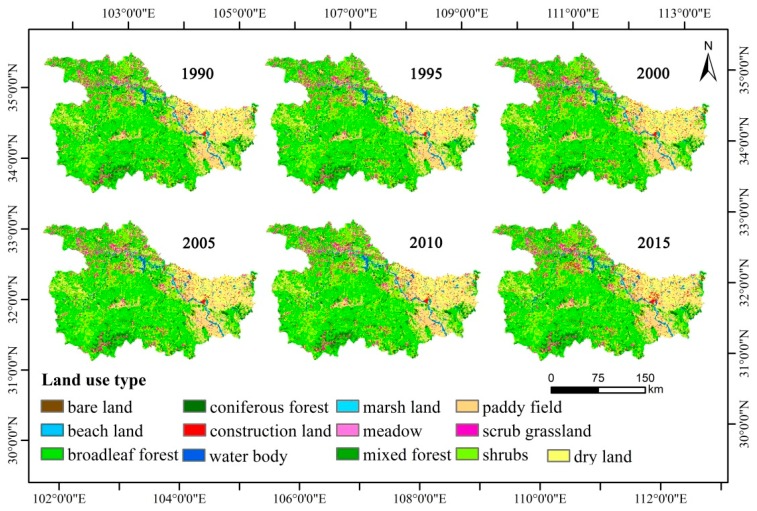
Land use types of the Exibei Region in 1990–2015.

**Figure 3:**
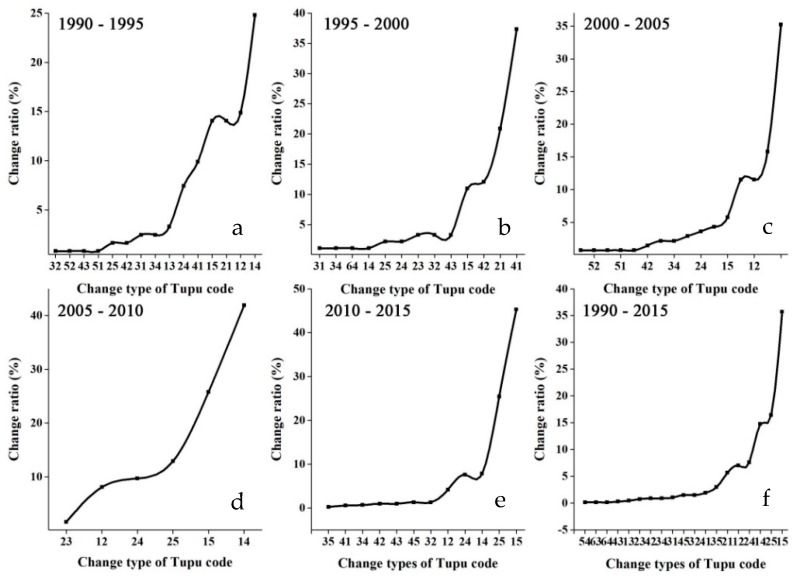
Change ratio of Tupu units of land use change during 1990–2015 in the Exibei region. (**a**): farmland; (**b**): forestland; (**c**): grassland; (**d**): water land; (**e**): construction land; (**f**): unused land. Tupu code was the transition type of land use changes, e.g., Tupu code *AB*, which meant that the land use type from type *A* at the former period to type *B* at the latter period.

Using geo-information Tupu to analyze the six years of land-use data that spans between 1990 and 2015, it was found that the main land use change was the increase in water body and construction land in the past two decades (Figure 2). As indicated previously, Tupu can indicate the direction and extent of land use changes (Figure 3). The transformation from farmland to construction land (Type code 15) was the major type of land-use change during 1990–2015, with an area converted totaling 4700 ha, which showed clustered distribution. However, among all land-use conversion types during 1990–2015, construction land (Type code 54) and unused land (Type code 64) transferring to water body exhibited a spatially dispersed pattern. Land-use change caused by the implementation of GGP accounted for 11.51%, 8.06%, 4.19% of overall land-use change during 2000–2005, 2005–2010, 2010–2015, respectively; and the separation degree of this transformation type also gradually increased during this time period as well indicating that the transformation was more dispersed. Southern and western parts of the region had larger areas of farmland transferred to forestland (Type code 12) and grassland (Type code 13).

**Figure 4:**
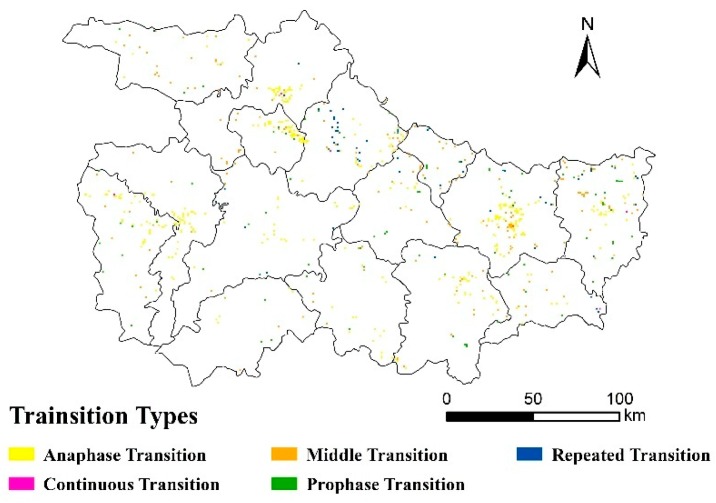
Pattern Tupu of land use change during 1990–2015 in the Exibei region. Anaphase transition type: land use type only changes in 2010–2015.; middle transition type: land use type only changes in 1995–2010.; repeated transition type: more than two kinds of land types changed in 1995–2010, and land use type remained the same in 1990 and 2015; continuous transition type: there were at least three land use types in 1990–2015, and land use type was different in 1990 and 2015.; prophase transition type: land use type only changes in 1990–2000.

To further analyze the change of land use pattern visually through Tupu, the land-use data of the year of 1990, 1995, 2000, 2005, 2010 and 2015, were combined in space and through time to build the classification system of land use change (Figure 4). Farmland transfer-out for forestland and grassland were main prophase transition type and anaphase transition type. The area of changing farmland transfer-out for forestland was 3500 ha during 2000–2015, which was nearly three times more than before the GGP; whereas, farmland transfer-out for grassland was the opposite. The prophase transition type of farmland transfer-out for forestland was mainly located in the south of the region namely Zhuxi, Fangxian, Shennongjia. The distribution of the anaphase transition type of farmland transfer-out for forestland was comparatively concentrated.

### 3.2. Spatio-Temporal Change of Ecosystem Services Value (ESV)

During 1990–1995, ESV showed a gentle upward trend, with a total increase of 0.43%. Following this increase was a general decline of ESV, the total value decreased by 1.21% between 1995 and 2000. The period 2000–2015 showed an increase of ESV up to 1.71%. Spatially, the regions along the banks of Han River had the greatest value. Between 1990 and 2015, areas with decreasing ESV per unit area occurred in the northwest and southeast region. Shiyan City in the northwest region showed a decline of ESV exceeding 6000 yuan/ha per unit ESV. In addition, the increase of per-unit ESV in the middle Exibei region, which is over 4000 yuan/ha, was the highest among all other areas of the Exibei region.

ESV was mainly composed of regulating services. The proportion of cultural services was relatively low when compared to other regions. The value of provisioning service, regulating service and cultural services both increased between 1990 and 2015; the regulating service value increased the most with an increase of 1.23%; however, the value of supporting services declined by 0.22%. From 1990–2015, hydrological regulation accounted for the largest proportion of the total ESV in the Exibei region. (Table 2)

### 3.3. The Effects of Supply and Demand Dynamics on the Ecosystem Services Scarcity Value (ESSV)

**Figure 5:**
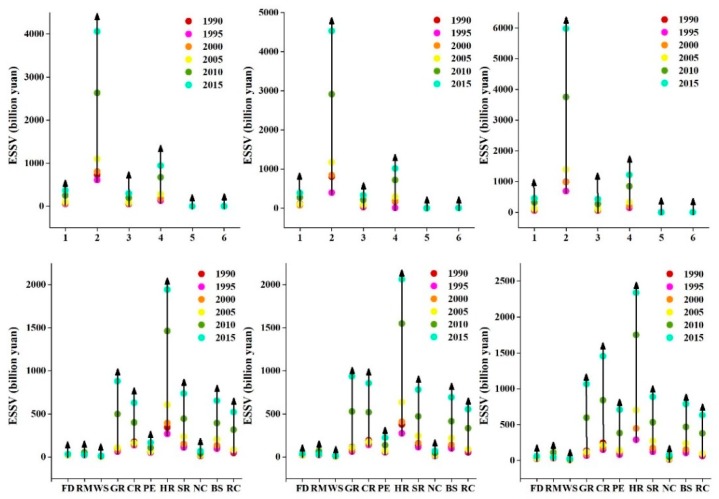
Ecosystem service scarcity values considering the simultaneous influence of supply and demand during 1990–2015 in the Exibei region. 1: farmland; 2: forestland; 3: grassland; 4: water body; 5: construction land; 6: unused land; FD: food production; RM: raw material; WS: water supply; GR: gas regulation; CR: climate regulation; PE: purify environment; HR: hydrological regulation; SR: soil retention; NC: nutrient cycling; BS: biodiversity services; RC: recreation and culture; ↑: rising trend of ecosystem services scarcity value.

Ecosystem services scarcity value quantifies the level of demand for ecosystem services. Under the change of reduced supply and rising demand, high ecosystem services scarcity value can indicate that the services become increasingly scarce relative to growing demand, and vice versa. Figure 5 showed the results of ecosystem service scarcity values (ESSVs) under the three simultaneous changes of supply and demand simultaneously changing in the Exibei region from 1990 to 2015. We found that overall trends in ESSVs under three scenarios were similar; ESSVs had increased for each and every ecosystem service type. Take ESSV under Scenario 2 as an example. From 1990–2015, due to an increase of demand and a reduction of supply, the ecosystem services scarcity value skyrocketed by 468.04%. The most valuable ecosystem services in 1990–2015 were hydrological regulation, gas regulation and climate regulation. Hydrological regulation increased in scarcity value nearly fivefold and remained the most valuable. With a sevenfold increase in scarcity value, gas regulation remained the second most valuable. Climate regulation scarcity value increased by 342.19%. Water supply was the least valuable services in 1990. It remained the same as the least valuable services in 2015 with only modest increases in scarcity value.

From 1990–2015, forestland was the dominant contributor to ESSV among land-use types in the Exibei region due to its high value-coefficients and large area. Despite declining in area by 1400 ha between 1990 and 2015, the total ESSV of forestland increased more than fivefold, a direct result steep rises in scarcity values for climate regulation, soil retention, nutrient cycling, and biodiversity services. Water body was the second most valuable with an increment of 490.54% from 1990 to 2015. The area of construction land increased, resulting in an increased ecosystem services cost. In general, the ecosystem services scarcity value increased across the board from 1990 to 2015, with the sharpest increases occurring in the western and central regions of the study area. The per-unit ESSV of these regions generally increased approximately 470% from 1990 to 2015.

### 3.4. Sensitivity Analysis for Ecosystem Service Value

We varied the ecological value coefficients of each ecosystem by plus and minus 50% to evaluate ecosystem service values by using sensitivity indices (Table 3). We found that the sensitivity indices of the total ESVs on ecological value coefficient was less than 1 for all the years analyzed (i.e., 1990, 1995, 2000, 2005, 2010 and 2015). It indicated that total ESV is inelastic with respect to ecosystem service value coefficient. Therefore, the ESV coefficients determined in this study had a good degree of fit and the estimation of ecosystem service value in this paper was reliable. The coefficient of variation fluctuated with a tendency of decreasing first, followed by an alternating increasing decreasing trend. It indicated that the balance of service values supported by land use types increased significantly before decreasing slightly, and then decreasing the balance again.

### 3.5. Analysis of Inhabitant Perceptions of Ecosystem Services

Community participation was necessary in order to determine the importance of these ecosystem services to local people [52]. The perceived decreases or increases in ecosystem services for the Exibei region were shown in Table 4. All the respondents told us that their livelihood mainly relied on forestry and agriculture. Our results showed that perceived improvement or deterioration of ecosystem services was fairly consistent with the changes in ESVs. Although the GGP and urban development have converted some farmland into other land-use types, the local agricultural landscape was large enough that the conversion had little effect on the crop cultivation of local residents who live on farmland. The main factor affecting food production was the varying weather conditions. Except for air purification and water supply and quality, other regulating and supporting services were not well perceived by the respondents. With the implementation of the GGP, the increase of forest area was uneven in space; thus, supporting services was increased, a change identified by respondents. The water system in the study area was large, and some areas established water system facilities, which increased the aquatic systems services. However, industrial development, particularly of the establishment of large factories to promote economic development, reduced the self-filtering capabilities of the ecosystem.

## 4. Discussion

### 4.1. Impact of GGP on Ecosystem Services

Land use change has altered the ecosystem services by influencing the processes and patterns of ecosystems [11]. According to land use transformation analysis, the ESV changes due to land-use changes were notable before and after the implementation of GGP. The transformation from water body to farmland was the major type of land-use change during 1990–2000, contributing to the decrease of ESV, while forestland transfer-out types mainly led to a decrease of ESV between 2000 and 2015 after the implementation of GGP. Regardless of the time periods, the increase of ESV could be attributed to farmland conversion to forestland and water body. The conversion of farmland to forestland and grassland could not lead to an increase in all ecosystem services at the same time [20], such as the decrease in food production value. Due to GGP, the regulating service value increased the most among all ecosystem service types with an increase of over 65% contributing to the growth of ESV. After the GGP, hydrological regulation accounted for the largest proportion of the increase of ESV.

Our results indicated that between 1990 and 2015, the total ESV in the Exibei region increased slightly by 0.91%; although the area of farmland, forestland and grassland were reduced by 2.61%, 0.47% and 1.41%. The increase of forestland and grassland was not significant with the implementation of GGP, because forestland was the dominant land type in this region, and close forest was also an important part of the GGP. In addition, as the water source of the Central Line Project of South-to-North Water Diversion, the Exibei region took great measures to protect water bodies, such as the construction of reservoirs and nature reserves. Thus, the increase of water land obviously derived the growth of regional ESV.

### 4.2. Impact of Supply and Demand Dynamics on the Ecosystem Services Scarcity Value

Ecosystem services (ES) are direct beneficial to humanity, as the goods and resources are provided by the environment and ES are crucial to economic development [53]. The availability of these benefits from the ecosystem is a precondition for ES demand [54]. ES demand can be defined as the level required or desired by human society or individual preferences for ES specific attributes, and is framed as consumption and desire in accordance with different ES categories [54]. The results showed that between 1990 and 2015, the population of the Exibei region grew by 12.16%, and the GDP per capita was about 37 times higher than before. In the meanwhile, ESSV increased by fivefold. The underlying cause of the increase of ESSV is the imbalance between ecosystem services supply and demand. There has been a decline in the supply for ecosystem services, while the demand for it has increased [55]. This is not surprising, because humans want to enjoy more ecosystem services with the improvement of living conditions, but the ecosystem does not have the capacity to provide enough services to meet the demand. When the influence of supply and demand dynamics was considered, assessing the total scarcity value of ecosystem services can effectively evaluate land management decisions, the scarcity value of forestland increased after the implementation of the GGP, and its annual growth rate during 2000–2015 was four times higher than that during 1990–2000.

Ecosystem services were classified as either private-good ecosystem services or public-good ecosystem services, according to whether they were more characteristic of public goods or private goods. In the study period, public-good type ecosystem services scarcity value increased more than that of private goods, particularly the scarcity value of recreation and culture. For private-good ecosystem such as food, scarcity can often be mitigated via other inputs [38]. In contrast, public-good type ecosystem services cannot easily be substituted by distant natural capital, technology, or other forms of capital [45,56]. Thus, the scarcity value of what is essentially unmet demand for these ecosystem services can become very large as the services become increasingly rare relative to the quantity demanded [45,57]. From 1990 to 2000, other than food production, water supply, gas regulation and climate regulation, the scarcity value of other services showed a downwards trend. After the implementation of the GGP, the scarcity value of various services increased due to the rise of population and income level. As demand for ecosystem services was driven up, the ecosystem services became scarcer. 

### 4.3. Impact of GGP on Local Communities Perception of Ecosystem Services

Humans tend to prefer provisioning services first within all the ecosystem service types, followed by regulating, supporting and cultural services [51,58]. Our results indicated that the dependence of rural livelihood on the provisioning services was the most important for both income generation and sustenance. The implementation of GGP largely changed the respondents’ life, although they all live in rural areas. With government support, some of the respondents that lived in the mountains and depend on the farmland on steep slopes for subsistence, moved to other communities with better living conditions and were provided other farmland. In addition, they perceived the importance of a provisioning service reflects the actual product demand and means of livelihood, which varied spatially [51]. For instance, Zijin developed characteristic forestry planting industry by planting economic seedlings such as *Cunninghamia lanceolata* (Lamb.) Hook. and *Camellia oleifera* Abel. Our study showed that the lack of recognition of regulating and supporting services was common among all respondents, except that they all thought highly of air purification [59]. However, the rapid industrialization in some villages has concealed the impact of GGP to improve air quality significantly, and has also been affecting water quality. Rural people in the area where nature reserves were established after the GGP think highly of cultural services, especially local culture and eco-tourism.

### 4.4. Sustainability of Ecosystem Services Management and Recommendations

Through GGP, the ecosystems social and economic benefits in the Exibei region have achieved the following. First, due to an extensive educational and public campaign, many locals are now aware of the need to preserve the local ecosystem. People have further recognized the importance of the country’s use of grain for ecological protection. At the same time, the enthusiasm for participating in the GGP and other ecological projects has been greatly enhanced. Second, the effectiveness of ecological forestry is evident. After more than 10 years of forestry, ecological engineering construction has greatly increased the forest vegetation in the Exibei region, which has improved the ecological environment and greatly enhanced soil and water conservation capabilities. Moreover, soil erosion has been significantly curbed, droughts, floods and other natural disasters have also decreased. Third, the construction of timber forests and economic forest bases has become an important source of continued income growth for farmers. The improvement of the ecological environment has effectively promoted agricultural production and food production growth. At the same time, the GGP has freed up a large amount of local rural labor force as they do not need to spend as much time as before for farming. Such a change has resulted in more workers engaging in industrial and services work. According to our results of field survey, most of the young people who were farming for generations have been liberated from the land after the GGP and have migrated to work, and their wage has become their main source of income. For farmers who have been engaged in farming, their dependence on land has been reduced due to the GGP, and they have been active in other industries such as animal husbandry and fishery during leisure time. 

Although the GGP has greatly changed traditional farming practices to no longer ineffectively cultivate the land, some problems remain in the management process. The amount of sloping farmland is also large in the Exibei region. Moreover, with the policies of supporting agriculture and benefiting agriculture constantly strengthening, the benefit of grain planting has improved coupled with the sharp rise in domestic grain prices in recent years. The income from participating in the GGP was obviously less than that from planting grain or renting farmland. Therefore, the enthusiasm among the farmers who depend on farmland highly was less when participating in the program, leading to a decrease in the effectiveness of the GGP. The consolidation of GGP was a result of inadequate care and management. In addition, the low level of forestry industrialization has proven difficult to improve both quality and efficiency wise. Therefore, we must strictly monitor the specific implementation of Grain for Green planting to ensure quality while guaranteeing quantity. We can improve by adhering to the following four aspects. First of all, we should increase the support for the policy of returning farmland to forests. Secondly, the investment in the later stage of GGP should be increased, and the subsidy standard for GGP improved as well. In addition, the activation management mechanism will improve the level of management of the project of GGP. Lastly, striving for funding and policy support from the government can accelerate the cultivation of the follow-up industry of returning farmland to forests.

## 5. Conclusions

The Exibei region, given its role as the ecological barrier in the geographical center of China and as the core water source area of the middle route of south-to-north water diversion project, requires careful management to ensure the sustainability of its environment. GGP, a large-scale ecological restoration project, has been implemented in the Exibei region for over two decades. Understanding its effectiveness is crucial in preserving ecosystem services and providing the ecosystem services needed. 

In this paper, products of remote sensing images and spatial statistics were used to assess land-use change in the Exibei region from 1990 to 2015 and to estimate the associated changes in the value of ecosystem services. The results showed that during 1990–2015, the most significant land use changes were that the construction land areas increased as well as the construction of several water conservancy projects and the establishment of nature reserves. With the implementation of GGP, the increase of ESV could be attributed to farmland conversion to forestland and water land. However, forestland transfer-out types mainly led to a decrease in ESV. When considering the impact of ecosystem services scarcity, the scarcity value of various services increased in spite of the implementation of the GGP, which was driven by the surging demand for ecosystem services. In particular, the scarcity value of public-good ecosystem services increased substantially due to the lack of substitutes. However, land-use changes do not fully explain the ecological benefits brought by GGP. In the future, we should focus on the impact of the GGP on regional natural conditions based on this study, and how these changes affect ecosystem services. Then, changes in ecosystem service values under the different implementation of GGP scenarios should be simulated to put forward the land optimization plan. Defining land use structure adjustment and its difference of influence on ESV due to the implementation of the GGP will provide decision reference for optimizing nature resources protection policy of China, which can effectively minimize trade-offs among multiple ecosystem services and promote regional sustainable development.

## Figures and Tables

**Table 1:** Price elasticities of supply and demand for private-good and public-good ecosystem services resulting in relative changes in scarcity value in the three scenarios analyzed in this study, and relative equations.

Scenarios	Elasticities				Price	
	Private-Good	Private-Good	Public-Good	Public-Good	Private-Good	Public-Good
	Supply	Demand	Supply	Demand		
Lowest	H:5.0	L:0.8	L:0.7	H:2.1	$P=1+\frac{2}{\left(e_{s}+e_{d}\right)}$	$P=1+\frac{1+e_{d}}{\left(e_{s}+e_{d}\right)}$
Medium	M:3.5	M:0.5	M:0.4	M:1.6		
Highest	L:2.0	H:0.2	H:0.1	L:1.1		

**Table 2:** Ecosystem service values in 1990–2015 (billion yuan).

Ecosystem Service Type		1990	1995	2000	2005	2010	2015
First Level	Second Level						
Provisioning service	Food production	15.12	15.11	15.10	15.09	15.06	14.91
	Raw material	15.11	15.11	15.12	15.10	15.10	15.02
	Water supply	2.74	2.96	2.44	3.01	3.15	3.42
Regulating service	Gas regulation	49.34	49.31	49.35	49.28	49.27	48.99
	Climate regulation	129.04	129.03	128.98	128.96	128.98	128.53
	Purify environment	41.71	41.81	41.56	41.82	41.90	41.89
	Hydrological regulation	162.40	164.52	158.38	164.87	166.10	167.80
Supporting service	Soil retention	56.81	56.80	56.82	56.77	56.78	56.55
	Nutrient cycling	5.14	5.14	5.15	5.14	5.13	5.10
	Biodiversity services	50.01	50.01	50.14	50.04	50.13	50.10
Cultural service	Recreation and culture	22.59	22.61	22.66	22.63	22.69	22.71

**Table 3:** Coefficient of sensitivity and variation for ecosystem service values.

Land Use Types	1990	1995	2000	2005	2010	2015
Farmland	0.0606	0.0602	0.0611	0.0600	0.0596	0.0585
Forestland	0.7427	0.7394	0.7481	0.7385	0.7362	0.7327
Grassland	0.0539	0.0536	0.0543	0.0535	0.0534	0.0527
Water body	0.1429	0.1470	0.1366	0.1483	0.1512	0.1566
Unused land	0.0002	0.0002	0.0001	0.0001	0.0001	0.0001
Construction land	0.0003	0.0003	0.0004	0.0004	0.0004	0.0006
Variation coefficient	1.7223	1.7141	1.7357	1.7122	1.7066	1.6990

**Table 4:** Perceived decreases or increases in ecosystem services for each village.

Ecosystem Service Types	Shi Hua	Zi Jin	Wen Fen	Majia Du
Food production	↓	－	－	↑
Raw material	－	↑	↑	－
Water supply	↑	↑	↑	－
Gas regulation	↑	↑	↑	↓
Climate regulation	↑	↑	↑	↓
Purify environment	↓	－	↓	↓
Hydrological regulation	－	↓	↓	－
Soil retention	↑	－	↑	↑
Nutrient cycling	－	－	－	↑
Biodiversity services	－	↑	－	－
Recreation and culture	－	↑	－	↑

↑: rising trend of ecosystem services value; ↓: declining trend of ecosystem services value; －: no significant change in ecosystem services value.
